# Hospital Clinics

**Published:** 1881-03

**Authors:** D. F. Wilson


### HOSPITAL CLINICS.

SERVICE OF

PROF. DONALD MACLEAN,

MEDICAL COLLEGE OF UNIVERSITY, MICHIGAN.

*Reported by D. F. Wilson.*

NOVEMBER 20th, 1880.

EXCISION OF THE TONSILS.—Little girl seven years of age. As the operation is not very painful, she was given no anæsthetic. The tonsil was firmly grasped with a pair of pronged forceps, and a clean incision of the tonsil made with a blunt-pointed bistoury having a concave edge. This operation, though simple, should never be made when there is active inflammation; so as to avoid hemorrhage. Dr. Maclean says: "I have only had two cases in which troublesome hemorrhage occurred."

DECEMBER 4th, 1880.

SIMPLE TUMORS.—A youth of 18 appeared before the class with two small cystic tumors; one just in front of the right ear, the other just below the lobe of the ear. They were removed without the use of an anæsthetic, and the wounds stitched with horse hair, which were removed seven days later. The contents were in a semi fluid state.

DECEMBER 15th, 1880.

MALIGNANT TUMOR.—This tumor commenced in the submaxillary gland, on the left side of a man, aged about forty-five. The operation was to remove one-half of the inferior-maxilla. An incision was first made through the middle of the lower lip, downward, to below the symphasis, then around under the body of the jaw, and up toward the ear. The bone was then sawed through at the symphasis. The jaw was next disarticulated so as to give some purchase on the tumor. A number of vessels were tied, and the disecting out of the tumor was completed, a part of carbolized cotton was put in the wound to preserve the contour of the face as much as possible. The figure of eight sutures were used to untie the lower lip, and interrupted sutures of silver were used to close the rest of the wound. The principal difficulty was to sever the temporal muscle. The facial lingual and other arteries were cut, but not much blood lost. The operation consumed about fifty minutes. The following day the patient was doing very well, and his temperature normal.

DECEMBER 11th, 1880.

OPERATION OF TREPHINING.—A man aged about 50, who had been suffering constantly and severely from neuralgia, came to the clinic for relief. Having chloroformed the patient, an incision of about two inches in length was made over the left side of the lower jaw. A button of bone was then cut out of the posterior part of the jaw, with a trephine about $\frac{1}{4}$ of an inch in diameter. A portion of the inferior-dental nerve was then excised, and the wound sewed up with horse hair. Four days after the operation, the patient appeared in clinic with the wound healed, and entiely relieved of his neuralgia.

